# Research on Reinforcement Learning-Based Autonomous Navigation and Obstacle Avoidance Methods for AGVs in Unknown Hospital Environments

**DOI:** 10.3390/s26113439

**Published:** 2026-05-29

**Authors:** Tianye Luo, Jing Hu, Bangcheng Zhang, Xinming Zhang, Shaoming Luo

**Affiliations:** 1 School of Mechatronical Engineering, Changchun University of Science and Technology, Changchun 130022, China; 2024200136@mails.cust.edu.cn (T.L.); hujing@cust.edu.cn (J.H.); zxm@cust.edu.cn (X.Z.); 2 Changchun Institute of Technology, Changchun 130103, China; zhangbangcheng@ccut.edu.cn; 3 School of Mechatronic Engineering and Automation, Foshan University, Foshan 528225, China; 4 Precision Machining and Special Machining Innovation Team, Guangdong Education Department, Foshan 528225, China

**Keywords:** reinforcement learning, AGV, obstacle avoidance, imitation learning

## Abstract

Reinforcement learning (RL) represents an effective approach for developing autonomous navigation and obstacle avoidance capabilities in hospital automated guided vehicles (AGVs). However, real-world adoption is challenged by the need for carefully designed reward functions, low sample efficiency, and slow convergence behaviour. To effectively address these issues, in this work, BEAGM-PPO, a reinforcement learning framework tailored for unknown hospital environments, was proposed. A reference model was initially employed to improve sample efficiency by directing the agent’s learning process. The reference model consists of expert demonstrations and policy derivation mechanisms. During the expert demonstration phase, human experts perform the required tasks and generate state-action pair datasets for training. During the policy derivation phase, demonstration data, behaviour cloning, and uncertainty estimation were used to derive the imitated expert policy. An ant colony optimization (ACO)-inspired pheromone mechanism and a memory replay strategy were incorporated to improve target-oriented action selection and supress unnecessary exploration. Experiments conducted in typical 3D simulation scenarios demonstrated that the proposed method achieved the highest arrival rate compared with baseline models. Moreover, the integrated imitation learning approach enables uncertainty estimation for both the policy and the model, while expanded training datasets further enhance performance. Overall, the results prove that BEAGM-PPO serves as a solid theoretical foundation for autonomous navigation in hospital AGVs.

## 1. Introduction

Recent progress in automation technology and AI has promoted the evolution of hospital logistics industry systems into intelligent and automated platforms [1]. The application of AGVs in hospital environments has become an important research and development direction for intelligent logistics systems. At the same time, AGVs are widely recognized as a crucial element in propelling the intelligent growth of the transportation sector [2].

Although AGVs have been widely used for many years, increasing attention is now being directed toward their obstacle avoidance performance. Autonomous systems are playing an increasingly important role in improving efficiency and reducing human-related errors [3]. Over the past few years, many researchers have adopted different approaches to autonomous navigation and obstacle avoidance decision-making. Visual cameras have been used to estimate depth for purposes like mapping and avoiding obstacles [4]. Additionally, this approach does not solely rely on visual devices but also on other types of sensors. For example, laser rangefinders can provide distance measurements for obstacle detection and 3D environment mapping or ultrasonic sensors can be directly integrated into obstacle detection systems. Yao et al. [5] proposed a 3D method based on LiDAR that can simultaneously track targets and map the static background. This method directly processes LiDAR point clouds to achieve accurate and stable multi-target tracking. Jing et al. [6] adopted a Bird’s-Eye View (BEV) space as a unified representation. Using a scene-level fusion (SLF) module, they employed LiDAR point cloud columns as depth queries to guide image features in performing depth alignment, thereby enhancing the ability to distinguish between foreground and background. This approach provides an effective solution for multimodal fusion in 3D object detection. Many researchers also utilize the Global Navigation Satellite System (GNSS) to provide continuous, reliable, and high-precision Position, Velocity, and Time (PVT) information for autonomous driving [7,8]. Nevertheless, AGVs cannot handle the hefty transmission systems needed for these sensors. Visual cameras are not limited by geographical conditions or location, as they are impervious to radiation and interference from signals [9]. Apart from being small, light, and energy-efficient, they also provide a wealth of environmental data [10]. Therefore, vision-only obstacle detection using cameras has emerged as a preferred solution for hospital AGVs.

Recent years have seen substantial advances and widespread adoption of RL technologies. For example, Yao et al. [11] proposed a 3-DOF method to improve the feasibility of trajectory generation and integrate advanced dynamic models; their research focuses more on outputting continuous control commands for the target heading angle. Through RL, robots can learn human-like decision-making strategies to solve real-world problems. The basic framework of RL is the Markov decision process, which enables agents to learn control strategies through trial and error [12]. The comparative advantage of this approach is that no extensive parameter tuning or precise environmental models are required, thus allowing the agent to autonomously accumulate experience and optimize its control strategy through continuous interaction with the environment [13]. However, RL continues to face challenges related to limited data efficiency, which results in unstable learning and slow convergence.

In the field of AGV obstacle avoidance, cameras or radar sensors are commonly used for environmental perception as input for reinforcement learning. Song et al. designed a multimodal deep reinforcement learning (DRL) method for indoor robot obstacle avoidance. The authors suggested a bilinear fusion module for environmental perception in order to extract complementary data from 2D radar data and photos, and then integrated this information into a deep Q-network to train control commands for mobile robots [14]. Tai et al. sampled raw laser ranging data and merged it with data from the laser rangefinder. The collected data serves as input for the DRL method, providing speed control commands to the robot and modifying the radar and camera fusion data to guide the motion output [15]. Qureshi et al. proposed a multimodal DQN model for biomimetic human interaction using a dual-stream Convolutional Neural Network (CNN) to extract grayscale and depth images. Then, deep neural networks were employed to derive multimodal feature representations [16]. In the aforementioned literature, radar and cameras are used as input sources. However, the heterogeneous data formats generated by different sensors render it challenging to fuse reliable and stable information from multiple sensors for obstacle avoidance [17].

In RL, a reference model is typically used to guide the model output actions and prevent it from deviating too far during training. The current mainstream approach is to select a pre-trained language model (PLM) that incorporates a large training corpus [18], such as web text or textbooks. Exceptional performance across a wide range of natural language processing (NLP) tasks has been demonstrated [19]. However, these models require further refinement before they can be applied to other fields [20]. Along these lines, in this work, the application of autonomous navigation and obstacle avoidance for AGVs in hospital corridors was systematically investigated. The considered framework addresses a continuous control problem involving a state space defined by sensor data, position, velocity, and other parameters. Existing PLM models are trained on the data in this work; they take word sequences as input and produce a probability distribution for the next word. Even with textual encoding, state-action representations cannot fully preserve the richness of the original information. At the same time, certain specific rules need to be incorporated to reflect real-world conditions. However, existing PLM systems are generic and cannot easily accommodate these customized rules. To this end, a reference model that outputs probabilities consistent with the action space distribution was developed, thereby aligning with the action space of the policy model.

To overcome the aforementioned problems, an enhanced reinforcement learning algorithm was introduced here. In particular, a camera as a means of environmental perception was used, serving as input. The BEVFormer model, combined with an improved PPO method (BEAGM-PPO) was implemented. In BEAGM-PPO, images are first processed using the BEVFormer model to detect obstacles. By combining the detected obstacle data with a stereo ranging method, the system can determine the obstacle’s position and then send this information to the agent. The agent uses the selected policy, along with ACO’s unique positive feedback mechanism and distributed exploration characteristics, to effectively explore output actions. At the same time, imitation learning and uncertainty policy were used to distil human prior knowledge into an estimated imitation expert policy, which serves as a reference model to guide the agent learning process and direct its action outputs. Next, based on the proposed adaptive importance-weighted priority experience replay mechanism, the collected experiences including state, action, and reward are placed into the experience pool. To reduce training time, BEAGM-PPO was trained in a simulated environment using the simulated values output by BEVFormer and then the agent was optimized by randomly sampling from the experience pool until the agent converged to the optimal state. Finally, a virtual hospital environment was created in Unity3D (2022.X) to test the BEAGM-PPO.

The following are the primary contributions of the paper:

- An integrated model combining expert reasoning with the simulation of experts’ prior knowledge was proposed as the reference model. By using imitation learning and uncertainty quantification methods to learn from experts’ prior knowledge, the agent’s output actions were guided;
- To accelerate the convergence of RL, an algorithm that uses the ACO pheromone model was proposed to guide agent decision-making. By leveraging the pheromone probability distribution of the ACO to guide actions, agents can significantly reduce aimless exploration. A priority replay mechanism with adaptive weights was also proposed to optimize the sample set and thereby accelerate convergence;
- Based on pedestrians’ spatial behaviour, the reward model for reinforcement learning was redesigned to penalize intrusions into pedestrians’ personal space, thereby enabling more effective interaction with pedestrians.

## 2. Related Work

### 2.1. Imitation Learning and Reinforcement Learning

Two extremely promising technologies used in autonomous driving are imitative learning (IL) and RL, both of which facilitate adaptive and human-like decision-making strategies. An effective supervised learning method is widely used in imitative learning; due to its simplicity and effectiveness, it has been widely adopted in autonomous driving research. For instance, modular driving systems and end-to-end autonomous driving systems both use imitation learning to generate control signals directly from raw sensor data [21], or a modular driving system that uses processed perception data to create a planned trajectory [22]. Nevertheless, imitation learning is inherently vulnerable to distribution shift issues. Moreover, compounding mistakes result in system failure because the training and inference distributions are not aligned. On the other hand, because RL depends on real-time interaction with the environment during training, it avoids this issue. RL has been used in several efforts to address difficult autonomous driving challenges, such as urban driving [23]. However, several well-known drawbacks of RL continue to impede its widespread application in practice. Even in simulated situations, RL is incredibly inefficient; yet, defining the reward function is challenging, and considerable effort is required to optimize parameters.

### 2.2. Learning with Human Prior Knowledge

Numerous works have suggested integrating human prior knowledge into RL frameworks to accelerate learning and enable risk-aware decision-making. Integrated human knowledge with RL may assist in solving autonomous driving tasks safely and successfully. Human prior knowledge can be introduced into RL systems in the form of expert demonstrations and task-related instructions. For instance, Chen [24] suggested the incorporation of a safety check module into RL-based control systems. This module encodes expert-defined safety criteria to direct the exploration process, remove risky behaviours, stop dangerous exploration, and speed up training. Additionally, the RL agent’s exploration is guided and the solution space is narrowed by human direction or demonstrations, which reduces needless interactions and speeds up the learning process. Additionally, Wu [25] presented a human-guided real-time learning approach that enables human specialists to give direction and assist in the training process in real time, allowing the RL agent to learn from both self-exploration and human supervision. A major limitation of this approach is its reliance on ongoing human guidance, which might overburden human experts. However, offline human demonstrations are easily accessible and do not necessitate interaction between the RL agent and human specialists. To enhance the performance of RL frameworks, it is therefore more beneficial to include demonstrations by human specialists.

### 2.3. Image-Based AGV Navigation Capabilities

CNN-based object detection algorithms may be incorporated into the PPO approach to produce more reliable picture navigation capabilities. The unmanned aerial vehicle (UAV) mentioned in Reference [26] used photographs captured by its onboard camera for autonomous navigation. To guide the quadcopter’s autonomous navigation, the YOLO model was applied to detect people in the photographs. The detection results were then transformed into a human-centered coordinate system. YOLO and a DQN algorithm were used to provide autonomous navigation for a lawnmower. YOLO was employed to identify certain landmarks in camera-captured pictures, and the DQN received the identification results directly for autonomous navigation [27]. Vehicles can avoid obstacles autonomously in a variety of situations by combining the DQN approach with the Faster R-CNN model [28]. Nevertheless, the processing of object detection information in existing approaches lacks the robustness required for the dynamic and unidentified AGV scenarios in hospitals examined in this work.

Despite recent progress, existing AGV navigation methods still face key limitations. Imitation learning suffers from distribution shift, while RL is constrained by low sample efficiency and complex reward design. Methods incorporating human knowledge often rely on online guidance or handcrafted rules, limiting scalability, and most fail to account for uncertainty in expert demonstrations. Perception frameworks combining RL and object detection frequently rely on simplified state representations, which are insufficient for dynamic and complex environments, while multimodal fusion introduces additional instability. Moreover, reference-model-based approaches are not well-suited for continuous control tasks.

To effectively address these issues, a BEAGM-PPO framework that integrates an uncertainty-aware reference model, an ACO-inspired exploration mechanism, and BEVFormer-based perception was proposed in this work, achieving more efficient and robust navigation.

## 3. Method

In this section, the kinematic model of the AGV is first presented, which serves as the control object in the AGV system. Since the method proposed in this work is essentially a reinforcement model, the relevant elements of reinforcement learning are defined in Section 3.2. Then, in Section 3.3, the proposed reference model, namely an integrated model with uncertain policy, is introduced. The following section describes improvements to the model.

Compared with existing methods employing weak interaction between imitation learning and RL, the proposed BEAGM-PPO framework introduces several key innovations in a tightly coupled and unified manner. First, an uncertainty-aware reference model was constructed to capture both policy and state uncertainty from expert demonstrations, enabling more robust guidance under distribution shifts. Second, an ACO-inspired pheromone mechanism was incorporated into the PPO framework to provide global exploration guidance, thereby reducing inefficient exploration and accelerating convergence. Third, a BEVFormer-based perception module was employed to generate structured bird’s-eye-view representations, enhancing spatial understanding in complex and dynamic environments. Finally, an adaptive prioritized experience replay strategy was designed to improve sample efficiency by emphasizing informative transitions during training.

### 3.1. Model of AGV Kinematics

**Figure 1:**
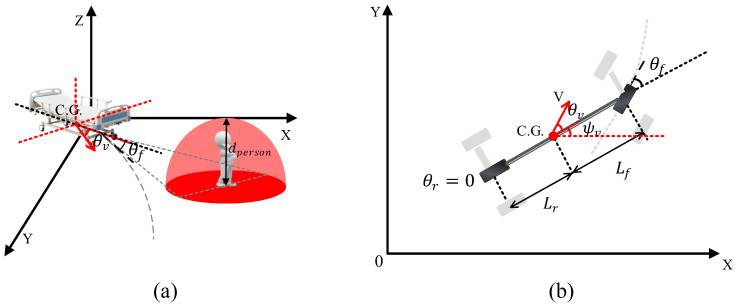
Kinematic Model of an AGV. (**a**) 3D model diagram; (**b**) AGV Planar Motion Model.

To understand policy applicable to real-world scenarios, the kinematic model of the AGV was incorporated into the controller of the simulation vehicle. The AGV kinematic model can be simplified to a planar model [29], as shown in Figure 1. The AGV model can be expressed by the following formula:

(1) $${\dot{X}}_{v}=Vcos(\theta_{v}+\psi )$$

(2) $${\dot{Y}}_{v}=Vsin(\theta_{v}+\psi )$$

(3) $$\dot{V}=a$$

(4) $$\dot{\psi }=\frac{V}{L_{r}}sin(\theta_{v})$$

(5) $$\theta_{v}=tan^{-1}(\frac{L_{f}tan\theta_{r}+L_{r}tan\theta_{f}}{L_{f}+L_{r}})$$

where ($X_{v}$, $Y_{v}$) represent the global coordinates of the center of gravity (C.G.), ψ is the heading in the global coordinate system (C.G.), V is the velocity of the center of mass in the global coordinate system, $L_{f}$ and $L_{r}$ represent the distances from the center of gravity to the front and rear axles, respectively, $\theta_{v}$ represents the angle between the direction of velocity V and the AGV centreline, a represents the AGV acceleration, and $\theta_{f}$ and $\theta_{r}$ represent the steering angles of the front and rear wheels, respectively, within the AGV frame. Generally, the rear wheel angle $\theta_{r}$ of a four-wheeled vehicle with front-wheel steering is zero.

### 3.2. Reinforcement Learning for AGV Autonomous Navigation and Obstacle Avoidance

In this section, states, actions, and reward functions in RL are defined. Then, the environment in RL was modelled. Specifically, in Section 3.2.2, the AGV autonomous obstacle avoidance problem as a Markov decision process was modelled.

#### 3.2.1. State and Action

The input state s(*t*) and the output action $a_{v}(t)$ were specified in order to more effectively handle the AGV obstacle avoidance problem, where t stands for time. In a mission, the input consists of the AGV current state, the destination state $s_{g}$ and image data $s_{o}$. The AGV status is determined by GPS.

(6) $$s_{v}=[X_{v}(t),Y_{v}(t),V,\theta_{f}(t),\psi (t)]$$

The target state, $s_{g}$ = [$X_{g}(t),Y_{g}(t)$], is either chosen in real time while the AGV is in a communicative state or determined before the mission starts. $s_{o}$ denotes images taken by the onboard camera, which are primarily used to detect obstacles and guide the AGV to avoid collisions. Within the inertial coordinate system, the destination state $s_{g}$ and the AGV state $s_{v}$ are values. The framework is intended to support wider deployment by maintaining applicability in dynamic and evolving situations. The definition of the input state s(t):

(7) $$s(t)=[s_{v}(t),s_{g}(t),s_{0}(t)]$$

(1) Reward function

As the AGV carries out its mission, the agent outputs action $a_{v}(t)$ based on the current state s(t). After executing the action $a_{v}(t)$, it receives an updated state s(t+1) and feedback from the environment. Environmental feedback is represented by the reward function output, $r_{v}$ and serves as the agent criterion for evaluating the current action $a_{v}$. Reward clipping is employed to quicken the agent’s convergence [30]. In the mission scenario described in this work, the AGV is expected to reach its destination. As a result, the reward function for whether the AGV arrives at its destination is set to:

(8) $$r_{arrived}=\left\{\begin{array}{l} +10,\mathrm{if}\mathrm{AGV}\mathrm{has}\mathrm{arrived}\\ 0,\mathrm{others} \end{array}\right.$$

(2) Comfort reward function

In scenarios where humans and robots interact, preserving pedestrian comfort requires the robot to avoid entering personal comfort zones without user engagement. The pedestrian’s personal space should not be invaded by the robot. Furthermore, Reference [31] states that a robot overtaking from behind has a lateral comfortable gap of 0.7 m. Pedestrians may experience discomfort if the distance is smaller than this. Thus, in this research, the reward function was adjusted to punish states in which the robot enters the pedestrian comfort zone based on the previously described requirements for pedestrian comfort.

Based on the relevant literature on pedestrian comfort [32], the pedestrian comfort zone Ω is defined by establishing a pedestrian coordinate system with the pedestrian as the origin and the forward direction as the x-axis. A semi-ellipse Ω with the pedestrian at its center and the maximum personal distance $d_{person}$ as its principal axis was used to depict the pedestrian comfort zone. Furthermore, the lateral comfort distance $d_{side}$ was taken as the short axis (taking into account just circumstances in which the pedestrian can see the AGV, $d_{side}$ = 0.7 m, $d_{person}$ = 1.2 m). Assume that ($x_{0},y_{0}$) are the robot’s coordinates in the pedestrian coordinate system. The robot’s entry inside the pedestrian comfort zone Ω may be determined using the exponential function $L_{\left(x_{0},y_{0}\right)}$. The robot has reached the pedestrian comfort zone if $L_{\left(x_{0},y_{0}\right)}$ is 1. The robot has not yet reached the pedestrian comfort zone, as shown by the $L_{\left(x_{0},y_{0}\right)}$ value of 0.

(9) $$L_{\left(x_{0},y_{0}\right)}=0,\frac{x_{0}^{2}}{d_{person}^{2}}+\frac{y_{0}^{2}}{d_{side}^{2}}\geq 1$$

(10) $$L_{\left(x_{0},y_{0}\right)}=1,\frac{x_{0}^{2}}{d_{person}^{2}}+\frac{y_{0}^{2}}{d_{side}^{2}}\leq 1$$

**Figure 2:**
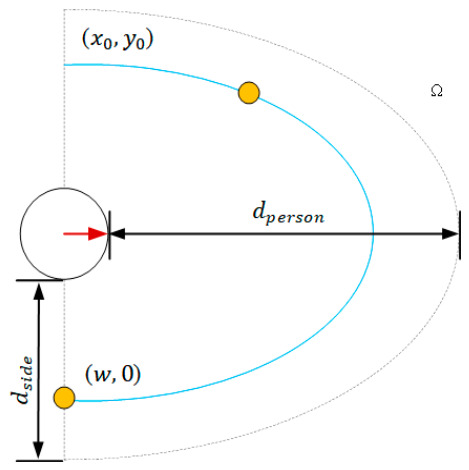
Pedestrian comfort distance.

The pedestrian will become uncomfortable if the robot moves into their comfort zone, that is $L_{\left(x_{0},y_{0}\right)}$ = 1, and the robot should be punished appropriately. The robot’σ location within the pedestrian comfort zone determines how severe the punishment is. The degree of penalty increases as the robot moves deeper into the pedestrian comfort zone. The robot is indicated in yellow as seen in Figure 2. It can be assumed that if the robot (yellow circle) enters the pedestrian comfort zone, the penalty would be the same at the robot’s coordinates as it would be on an ellipse with the same major-to-minor axis ratio. Put differently, ($x_{0},y_{0}$) and (w, 0) have the same penalty as every point on the blue ellipse in Figure 2 has the same penalty term. Closer robot-pedestrian proximity is associated with a higher level of pedestrian discomfort. Therefore, this penalty value can be represented using an exponential function, where the maximum value of the penalty is $r_{q}$. Thus, the penalty function in terms of *w* is given by:

(11) $$R=-r_{q}(e^{-(w-d_{\mathrm{side}})}-1)$$

The blue ellipse’s short axis is denoted by *w*.

(12) $$w=\sqrt{\frac{\left(x_{0}^{2}\times d_{\mathrm{side}}^{2}\right)}{d_{\mathrm{person}}^{2}+y_{0}^{2}}}$$

(3) Basic Safety Reward

This reward primarily evaluates the distance between AGVs and pedestrians. Positive reinforcement is provided when the distance to the AGV ahead exceeds the comfort distance. Negative reinforcement is applied when the distance to the AGV ahead falls below the comfort distance. The goal of this design is to incentivize AGVs to keep a safe distance and refrain from upsetting pedestrians [33]. The reward function is shown below.

(13) $$r_{1}=\left\{\begin{array}{l} 1.0,\mathrm{if}\mathrm{CS}-\mathrm{CD}>0.7\\ -5.0\times R,\mathrm{if}\mathrm{CS}-\mathrm{CD}\leq 0.7 \end{array}\right.$$

where CS represents the current distance and CD represents comfort distance.

(4) Transportation Efficiency Reward

Speed deviation, defined as the difference between the pedestrian’s speed and the AGV’s current speed, together with a time-step penalty, forms this reward function. It directs the AGV to keep its speed around the desired speed by imposing a negative reward for speed deviations while taking time costs into account to promote efficient traffic flow. The reward function is given by the following formula.

(14) $$r_{2}=-1.0\times |V-V_{\mathrm{pedestrain}}|-\Delta t$$

where V stands for the AGV speed and $V_{pedestrian}$ represents the pedestrian’s speed. In this work, it was set to 1.2 m/s. Δt denotes the time step penalty.

(5) AGV Path Deviation Reward

In this work, it is necessary for the AGV to follow the road’s centerline. Center lane deviation is 0. The left side of the front is negative; this value decreases as the offset increases. The positive side of the front is on the right; this value increases with the offset. This reward function is given by the following formula.

(15) $$r_{3}=e^{-|x^{2}|+1}$$

This function was used to take advantage of its properties: its graph is axisymmetric, and the function reaches its maximum value at 0, with the value increasing as the input increases. A smaller function value indicates a reduced score; therefore, this function penalizes lane departure. Given the importance of lane departure to the overall reward mechanism, $r_{3}$ is the penalty value. Lane departure has a maximum threshold of $l_{thr}$. When $l_{thr}>2m$, the AGV deviates too far from the lane and exits it, immediately ending the training. If the offset threshold exceeds an appropriate range, AGV collisions may occur, negatively affecting training outcomes. Excessively low thresholds increase computational and training demands while making robust model learning more difficult. $r_{4}$ is the deviation penalty for the AGV. The reward value is −5 when the AGV strays from its lane and goes beyond the threshold $l_{thr}$, which serves as a strong penalty for lane deviation.

(16) $$r_{4}=\left\{\begin{array}{l} -5,\mathrm{if}l>l_{thr}\\ 0,otherwise \end{array}\right.$$

(6) Speed Reward

In accordance with hospital regulations, a speed limit was set for AGVs. Any vehicle exceeding this limit will be subject to penalties. The penalty value is $r_{fast}$ and the threshold was set to 1.5 m/s. When the vehicle speed exceeds the threshold, $r_{fast}$ = −10. Otherwise, the case is 0.

(17) $$r_{fast}=\left\{\begin{array}{l} -10,\mathrm{if}V>1.5\;m/s\\ 0,otherwise \end{array}\right.$$

(7) Penalties for Violations

When an AGV is in motion, factors such as excessive speed, slow braking, and pedestrians suddenly changing lanes can all lead to collisions. If a collision occurs during training, the current test run is immediately terminated and the next simulation is started. $r_{5}$ is the penalty for an AGV collision. If a collision occurs, the reward value is −100. Otherwise, it is 0. Collisions directly affect pedestrian safety. The AGV must not hit anything in order to complete the experiment.

(18) $$r_{5}=\left\{\begin{array}{l} -100,\mathrm{if}\mathrm{collision}\\ 0,otherwise \end{array}\right.$$

(8) Risk of relative motion

(19) $$r_{6}=\left\{\begin{array}{l} -1\times \frac{1}{d_{p}-0.7+\varepsilon },d_{p}<1.5\;m,d_{\left(p\right)}<0\\ 0,otherwise \end{array}\right.$$

where $d_{p}$ represents the real-time distance between the AGV and the pedestrian, $d_{\left(p\right)}$ denotes the rate of change in the distance between them. The value of 1.5 was selected for the safety distance threshold, 0.7 was the minimum allowable distance, and ϵ was used to prevent the denominator from becoming zero. The purpose of this term was to ensure that when a pedestrian actively approaches the AGV, causing the relative distance to decrease, the AGV still receives negative feedback in advance, thereby learning a more conservative and safer avoidance policy.

Consequently, the reward function has the following definition.

(20) $$r_{v}(s(t),a_{v})=r_{arrived}+r_{fast}+r_{1}+r_{2}+r_{3}+r_{4}+r_{5}+r_{6}$$

#### 3.2.2. Markov Decision Process

Considering the current time step’s state s(t), the agent chooses the action $a_{v}(t)$ to carry out while the AGV is working on a job. The state s(t) changes to s(t + 1) and is rewarded by the environment after executing an action. The process by which an agent selects an action can be considered to have Markovian properties. This process can be referred to as a Markov decision process [34].

(21) $$\left\{\begin{array}{l} MDP=S_{v},A_{v},R_{v},\mathrm{state}\mathrm{transition}\mathrm{probability}\\ S_{v}=[s(1),s(2),\ldots ,s(t),\ldots ]\\ A_{v}=a_{v}(1),a_{v}(2)\ldots ,a_{v}(t),\ldots ]\\ \mathrm{state}\mathrm{transition}\mathrm{probability}=p(s(t+1),R_{v}(t+1)|s(t),a_{v}(t))\\ R_{v}(t+1)=r_{v}(s(t),a_{v}(t)) \end{array}\right.$$

where $S_{v}$ denotes the state space and s(t) denotes the state at the *t*-th time step. $A_{v}$ is the set of action spaces, and $a_{v}(t$) denotes the action performed at time step t. The likelihood of moving to the new state s(t + 1) and getting a reward $r_{v}(t+1)$ following the execution of action $a_{v}(t)$ in the current state s(t) is known as the *state transition probability*. $R_{v}$ is the reward function. The reward received after carrying out action $a_{v}(t)$ in state s(t) is denoted by $r_{v}(t+1)$.

### 3.3. Reinforcement Learning Demonstrated by Experts

To increase training sample efficiency for RL—for example, in safety and risk aversion—various policies have been incorporated. Many works in the literature have proposed RL methods that incorporate human prior knowledge [24]. Inspired by this approach, this work established a reference model for an integrated approach based on human understanding and proposes incorporating imitation learning to guide the agent actions in RL amidst the uncertainty of expert policies. As expert policies are difficult to obtain directly, behaviour cloning based on expert demonstrations is often adopted to learn approximate policies [35]. This method can only generate deterministic policies that estimate action points. Regularizing the action distribution of stochastic RL rules also requires an action distribution.

#### 3.3.1. Policy Uncertainty

The uncertainty present in human (expert) behaviour, which implies that an individual may produce many possible behaviours in the same state, was first taken into account in order to arrive at a stochastic expert policy. We refer to this uncertainty as policy uncertainty. It stems from the inherent randomness underlying human experts’ actions. Thus, in order to take this uncertainty into account, the probability distribution of the parameters of the model was used to determine the parameters of the output rather than those of the deterministic model. Furthermore, the action was assumed to follow a Gaussian distribution, that is, $a_{t}\sim N(\mu_{\theta }(s_{t}),\sigma_{\theta }^{2}(s_{t}))$. Next, the expert imitative policy was trained using the maximum likelihood method. This is the same as minimizing a Gaussian distribution negative log-likelihood (NLL) [36].

(22) $$L(\theta )=\underset{(a,s)\sim D^{E}}{E}[\frac{\log{\hat{\sigma }}_{\theta }^{2}(s_{t})}{2}+\frac{{\left(a-\hat{\mu }(s_{t})\right)}^{2}}{2{\hat{\sigma }}_{\theta }^{2}(s_{t})}+c]$$

where a is θ parameter of the policy network, ${\hat{\mu }}_{\theta }$ and ${\hat{\sigma }}_{\theta }^{2}$ are the predicted mean and variance, respectively, and c is a constant.

#### 3.3.2. Model Uncertainty

The predicted mean and variance of our policy predictions are still ambiguous and untrustworthy for data that was not part of the training dataset, despite the fact that a stochastic expert policy has been generated. We refer to this uncertainty as model uncertainty. This is because some areas of the state space lack training data. In our approach, estimating the model’s uncertainty is essential to imitating expert policy. because RL agents frequently come across data items that are absent from the demonstration dataset. Therefore, it is necessary to quantify the confidence level for states outside the distribution. Several methods have been proposed to estimate this uncertainty, such as the dropout Monte Carlo method [37] and the deep ensemble method [38]. The deep integrated method was employed in this work due to its greater processing efficiency and ease of implementation. A collection of M networks (randomized policies) was used, which were trained using Equation (22) with different random initializations and data orders. A Gaussian mixed distribution was applied to aggregate the outcomes of all networks (where $\theta_{i}$ is the parameter of the ith network). The mixture mean and variance can be calculated as:

(23) $$\mu_{\pi^{E}}(s_{t})=\frac{1}{M}\sum_{i=1}^{M}{\hat{\mu }}_{\theta_{i}}(s_{t})$$

(24) $$\sigma_{\pi^{E}}^{2}(s_{t})=\frac{1}{M}\sum_{i=1}^{M}{\hat{\sigma }}_{\theta_{i}}^{2}(s_{t})+[\frac{1}{M}\sum_{i=1}^{M}{\hat{\mu }}_{\theta_{i}}^{2}(s_{t})-\mu_{\pi^{E}}^{2}(s_{t})]$$

The integrated model, which incorporates both policy uncertainty and model uncertainty, may be thought of as a Gaussian expert policy with mean $\mu_{\pi^{E}}(s_{t})$ and variance $\sigma_{\pi^{E}}^{2}(s_{t})$.

### 3.4. PPO Method

The deep reinforcement learning method, known as Proximal Policy Optimization (PPO), was developed by Schulman et al. [39]. An actor–critic framework for policy optimization is employed in this framework. The PPO algorithm interacts with the environment to collect data, and then uses multiple rounds of mini-batch stochastic gradient ascent to optimize the objective function.

(25) $$L(\theta )={\hat{Ε}}_{t}[\min(r_{t}(\theta )]A_{t},\mathrm{clip}(r_{t}(\theta ),1-\varepsilon ,1+\varepsilon )A_{t})]$$

**Figure 3:**
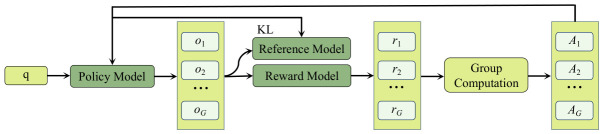
The GRPO model.

Due to the real-time application requirements of deep reinforcement learning, PPO models must be immediately deployed after training to begin a new round of data collection. Therefore, a network architecture with lower computational requirements is needed to quickly adapt to new environments. Furthermore, unlike supervised learning, deep reinforcement learning suffers from poor data stability, making it difficult to clearly distinguish between the training set and the test set. Therefore, network architectures with too many parameters or excessive computational resource consumption should not be used to avoid overfitting. In autonomous driving tasks, the use of historical data is particularly critical for the ongoing decision-making process. However, autonomous driving involves extremely frequent decision-making. Overloading the state vector with historical features may increase model complexity and hinder effective network training. To address this issue, we draw on the approach of the GRPO model [40]. The improved structure is shown in Figure 3.

This method estimates the replacement value function directly using intra-group relative advantages, samples multiple action outputs for the same state, and calculates the advantage function using the intra-group average reward as a baseline, effectively reducing video memory usage. The reliance on independent evaluators is eliminated through intra-group comparison. In this way, the number of forward and backward passes required during training can be reduced, thereby significantly lowering the computational resource requirements. By comparing actions within the same group instead of assigning individual absolute scores, this framework improves the model’s interpretability of action quality. At the same time, the ability to easily scale computations that leverage the group’s strengths simplifies the reward evaluation process. To ensure that the fitting results are optimized, the network loss function is derived as follows.

(1) The population mean of the reward is calculated.

(26) $$\mu_{G}=\frac{1}{G}\sum_{t=1}^{G}R(a_{t})$$

Standard deviation of rewards.

(27) $$\sigma_{G}=\sqrt{\frac{1}{G}\sum_{t=1}^{G}(R(a_{t})-\mu_{G}{)}^{2}}$$

(2) The relative reward for the group is calculated. For each action $a_{t}$ in the group, its relative reward within the group is calculated.
  (28) $$A_{i}^{G}=\frac{R(a_{t})-\mu_{G}}{\sigma_{G}+\varepsilon }$$

In this context, ε is a smoothing term, commonly used to avoid having a zero in the denominator.

(3) Compute the loss function. For each action $a_{t}$, compute the loss based on $A_{i}^{G}$.
  (29) $$L_{i}(\theta )=-log\pi_{\theta }(a_{t}|s_{t})\times A_{i}^{G}$$

(4) The objective function is updated.

The objective function is constructed by averaging the losses of all actions within the group and adding a KL divergence penalty term.

(30) $$J_{\text{AGM-PPO}}\theta =E[\frac{1}{G}\sum_{i=1}^{G}L_{i}(\theta )]-\beta D_{KL}(\pi_{\theta }||\pi_{ref})$$

(31) $$D_{KL}[\pi_{\theta }||\pi_{ref}]=\frac{\pi_{ref}}{\pi_{\theta }}-log\frac{\pi_{ref}}{\pi_{\theta }}-1$$

(8) Gradient Update. Gradient of the objective function.

(32) $$\nabla_{\theta }J_{\text{AGM-PPO}}=E\frac{1}{G}\sum_{i=1}^{G}[\nabla_{\theta }log\pi_{\theta }(a_{t}|s_{t})gA_{i}^{G}]-\beta \nabla_{\theta }D_{KL}$$

Parameter updates.

(33) $$\theta \leftarrow \theta +\eta \nabla_{\theta }J_{\text{AGM-PPO}}$$

### 3.5. Framework of the BEAGM-PPO Method

**Figure 4:**
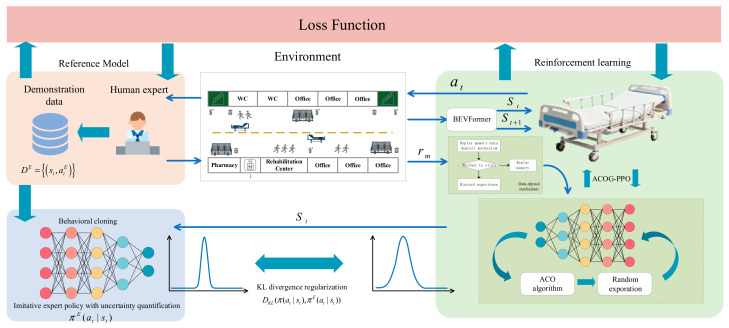
Overall structure of BEAGM-PPO.

Figure 4 depicts the structure and data flow of the BEVAGM-PPO approach. The proposed approach differs from PPO in several important ways: (A) the CNN module was replaced with BevFormer to extract image data [41], which is a model with superior detection capabilities; (B) an expert policy for imitation is derived by the reference model using behavioural cloning and uncertainty estimation; (C) a priority experience pool with adaptive weights is proposed; and (D) the ACO algorithm is introduced to determine the optimal action. The spatial position is then determined using stereo triangulation from the dual cameras, combined with the parallax of the obstacles, to measure the distance between the AGV and the obstacles [42].

In particular, AGV transmits the image $s_{0}$ into the BevFormer model after gathering data s(t) via a camera. After extracting features from the image, the BevFormer model delivers obstacle information $s_{0}^{\prime }$. After determining the distance between the obstacle and the AGV using stereo ranging technology and combining this with the obstacle information, the prediction network receives the state $s^{\prime }(t)$ as input. Based on the state $s^{\prime }(t)$, the prediction network incorporates the pheromone mechanism of ACO to select the current possible action with the goal of maximizing rewards. The optimal action is determined by minimizing the number of ineffective explorations in the environment, and then the optimal action $a_{v}(t)$ is executed. Following the execution of action $a_{v}(t)$, the environment will return the reward $r_{v}(s(t),a_{v}(t))$ and the new state s(t + 1). Next, it is determined whether the current experience $r_{m}=[s(t),a_{v}(t),r_{v}(s(t),a_{v}(t)),s(t+1)]$ record is kept in memory depending on the data storage method; if not, it is discarded. The percentage of actions linked to each kind of experience in the memory will shift once it is added, ensuring that every kind of action experience has a suitable sampling probability. The target network is then fed m sets of events that were randomly selected from the replay memory. The error is determined using the loss function. The prediction network is optimized using gradient descent based on the error. After extensive training and optimization, an agent capable of solving the problem is finally obtained.

### 3.6. Priority-Based Experience Replay with Adaptive Importance Weights

In RL, an experience replay mechanism is introduced to improve sample efficiency and eliminate data dependencies. During an agent exploration process, existing experiences are stored in an experience pool. Once the experience pool is full, new experiences will overwrite the old. The proposed approach improves the reuse rate of experiences, accelerates convergence, and enhances accuracy as the replay memory is progressively updated. However, if all the experiences generated during the agent’s exploration process are placed into an experience pool for later use, drawing on these experiences for training—given their low value in the early stages of training—will hinder convergence. For example, the experience pool will store a large number of intermediate state experiences with zero reward values, as well as a small number of terminal step experiences with non-zero reward values. Therefore, the BEAGM-PPO algorithm introduces an empirical filtering mechanism and proposes a method with adaptive importance weights. Adaptive importance weighting enables the model to focus on more valuable samples, thereby improving training speed.

In the transformation pool, each sample *i* has a priority $p_{i}$. The error is positively correlated with $p_{i}$ and *i.* The priority $p_{i}$ for sample *i* is calculated as follows.

(34) $$p_{i}=|error_{i}|+\varepsilon$$

where $p_{i}$ represents the priority of sample *i*; ε is a very small positive number used to ensure that each sample has a chance of being selected.

The following formula can be used to get the sampling probability $p_{\left(i\right)}$ for each sample.

(35) $$P_{\left(i\right)}=\frac{P_{i}^{a}}{\sum_{k}P_{k}^{a}}$$

where a is a parameter between 0 and 1 used to control the degree of influence of the priority. This ensures that samples with larger errors are given priority during training.

To address the bias introduced by priority sampling, importance sampling weights are introduced to adjust the gradient of the sampled samples. The formula is as follows.

(36) $$\omega_{i}={\left[N\times P_{\left(i\right)}\right]}^{-\beta }$$

The weights in the above formula can be normalised to ensure greater stability during training.

(37) $$\omega_{i}^{*}=\frac{{\left[N\times P_{\left(i\right)}\right]}^{-\beta }}{{max}_{i}\omega_{i}}$$

where N represents the capacity of the experience replay pool, and *β* is a number between 0 and 1 used to control the strength of the weights. Traditionally, *β* is a fixed constant; in this work, it increased as the number of iterations increased, as shown below.

(38) $$\beta =\{\begin{array}{c} \beta +\alpha \\ 1 \end{array}\begin{array}{c} \beta \leq 1\\ \beta >1 \end{array}$$

where *a* is a constant, and β is used to adjust the importance sampling; during training, it increases linearly from a small value to 1. When the β value is small, the algorithm tends to select samples with higher priority, thereby focusing more on samples that have a significant impact on the algorithm training. When the β value is large, the algorithm tends to select samples more uniformly to ensure that it can learn strategies from a diverse set of samples. The proposed mechanism improves the effectiveness of prioritized sampling and accelerates learning from important samples. Toward the end of training, sampling bias is reduced to improve learning stability.

### 3.7. ACO-Guided BEGM-PPO Algorithm

ACO [43] is a bio-inspired algorithm that mimics the foraging behaviour of ant colonies in nature. Ants exchange information through the transmission and release of pheromones, using a fully distributed approach to communicate. At time t, ant k is at node i and selects the next node j based on the pheromone concentration $A_{ij}(t)$ and the magnitude of the guidance signal $Z_{ij}(t)$. With probability p(p∈[0,1]), the ant selects the node j that maximizes ${A_{ij}(t)}^{\varsigma }{Z_{ij}(t)}^{\beta }$; with probability 1 − p, it randomly selects the next node.

(39) $$P_{ij}^{k}t=\left\{\begin{array}{l} \frac{{\left[A_{ij}(t)\right]}^{\varsigma }{\left[Z_{ij}(t)\right]}^{\beta }}{\sum_{l\in allowed}{\left[A_{ij}(t)\right]}^{\varsigma }{\left[Z_{ij}(t)\right]}^{\beta }},j\in allowed_{k};\\ 0,otherwise. \end{array}\right.$$

(40) $$\eta_{ij}(t)=\frac{1}{d_{jg}}$$

here ς is the pheromone-inspired factor, ${allowed}_{k}$ is the set of the next nodes that the kth ant can currently reach, β is the expected heuristic factor, and $d_{jg}$ is the Euclidean distance from node j to node g.

The updated formula for the ACO global pheromone is:

(41) $$A_{ij}(t+1)=(1-p)A_{ij}(t)+\Delta A_{ij}(t)$$

(42) $$\Delta A_{ij}(t)=\sum_{k=1}^{M}\Delta A_{ij}^{k}(t)$$

(43) $$\Delta A_{ij}^{k}(t)=\left\{\begin{array}{l} \frac{Q}{L_{k}(t)},\mathrm{the}\mathrm{path}\mathrm{taken}\mathrm{by}\mathrm{ant}k\mathrm{through}(i,j)\\ 0,otherwise \end{array}\right.$$

where p(p∈[0,1]) is the evaporation coefficient of the pheromone. ${∆A}_{ij}(t)$ is the total amount of pheromone left by all ants between the two nodes at time t. M is the total number of ants, Q is the pheromone intensity, ${∆A}_{ij}^{k}(t)$ is the pheromone trail left by ant k as it travelled from node i to node j during iteration t, and $L_{k}(t)$ denotes the total length of the path travelled by ant k during iteration t.

**Figure 5:**
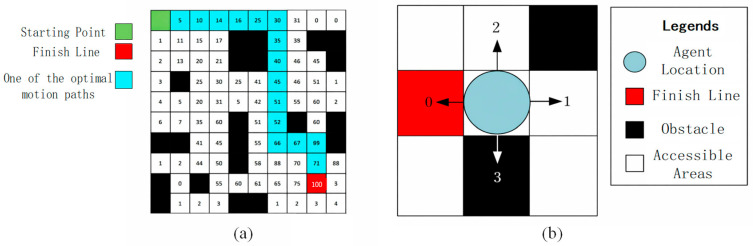
ACO Algorithm (**a**) ACO Algorithmic Pheromone Model (**b**) Action selection.

The pheromone mechanism of ACO is the primary method guiding its convergence, as shown in Figure 5a. Its update mechanism works as follows: after all ants have completed their respective iterations in a single cycle, a global pheromone update is performed. The pheromone renewal process consists of two steps: pheromone volatilization and pheromone superposition. The release of pheromones can filter out the chaotic pheromone trails left behind during the initial exploration phase of the ant colony. Pheromone superposition allows more pheromones to be superimposed onto the ideal action, promoting rapid convergence during reinforcement learning training.

First, the pheromone mechanism in ACOs provides agents with information about global action trends. It allows the system to comprehensively consider both the current PPO estimate and the historical action information encoded in pheromone concentrations when selecting actions, thereby increasing the likelihood of exploring in the direction of potentially high-quality actions. This not only effectively reduces the number of times the agent engages in blind exploration but also accelerates the algorithm convergence process. Second, the multi-agent collaborative exploration capabilities of ACO enable distributed sampling of the environment. Through continuous pheromone accumulation and adaptation, the framework enhances environmental exploration and reduces PPO’s tendency to become trapped in local optima. In addition, the seamless integration of the empirical filtering mechanism proposed in this work with ACO further improves sample quality. The enhanced learning capability of BEGM-PPO allows the model to develop better motion strategies, thereby comprehensively improving both algorithm performance across key metrics such as convergence speed, motion strategies, and algorithm stability.

### 3.8. ACO Algorithm-Based Action Selection Policy

After performing an action, the PPO algorithm transitions to the next state, where it receives feedback from the environment and performs the next action. In conventional RL frameworks, action selection relies on the policy model and incorporates random exploration to improve learning. Random exploration can generate actions that differ from those of neural networks, seeking out a wider range of possible actions to enrich the network parameters. However, random search is generally less efficient, as shown in Figure 5b. In a rasterized map, a four-neighbourhood search method is used. When the agent reaches the current position, selecting Action 1 or Action 2 constitutes an invalid exploration, and selecting Action 3 would result in a collision with an obstacle, which is an illegal exploration; only selecting Action 0 constitutes a valid exploration. If random exploration is used, there is only a 25% chance of selecting the correct exploration action. When selecting an action at each step, an agent typically has only one or two valid options to explore. Therefore, when using random exploration during the learning process, the agent is likely to select ineffective exploration actions. Excessive numbers of ineffective iterations are not only time-consuming but also hinder network convergence, which may result in inaccurate outputs from BEGM-PPO during the early stages of training.

In ACO, pheromones guide subsequent decisions through the continuous accumulation and updating of historical values, reflecting the potential optimal direction from a global perspective. In addition, the distributed exploration strategy of the ACO algorithm is conceptually aligned with the experience replay mechanism utilized in BEGM-PPO. Multiple ants conduct their own local explorations within the environment, communicating movement information through the accumulation of pheromones. This fully distributed search approach can significantly improve search efficiency. Therefore, the refinement of the pheromone matrix can be viewed as a distributed method of accumulating experience, whose mathematical form is:

(44) $$P(a_{t}|s_{t})=A{\left(s_{t},a_{t}\right)}^{\alpha }\times Z{\left(s_{t},a_{t}\right)}^{\beta }$$

where Z represents the heuristic function. By adjusting the weights *a* and β, the pheromone mechanism can dynamically balance the current global action selection trend and avoid local optima. In ACO, ants select their actions based on differences in pheromone concentration in their local neighbourhoods. Suppose the agent’s current node coordinates are (*x*, *y*). In its four neighbouring regions, the pheromone concentration is significantly higher than at the node itself, and the node with the highest concentration is its next ideal action choice node, denoted as $\left(x^{\prime },y^{\prime }\right)$. In the ACO-Improved PPO algorithm, the mechanism for selecting the next action is as follows.

(45) $$a(x^{\prime },y^{\prime })=max\left[A(i,j)\right]$$

(46) (i,j)=(x-1,y)or(x+1,y)or(x,y-1)or(x,y+1)

where $a(x^{\prime },y^{\prime })$ represents the next action selected by the ACO and A represents the pheromone concentration and (*i*, *j*) represents the coordinates of its neighbourhood. In the BEGM-PPO algorithm, the introduction of an ACO to replace some of the random decision-making significantly reduces the number of ineffective explorations during the early stages of training when the agent selects actions. In the BEAGM-PPO algorithm, ACO and PPO are explored in parallel. As the ants undergo continuous iterations, pheromones are distributed along the exploration paths through these iterations, while PPO updates the parameters of the target network based on the loss function.

The proposed BEAGM-PPO algorithm employs ACO to replace some stochastic decisions, significantly reducing unnecessary exploration during the early stages of training. In the BEAGM-PPO algorithm, ACO and PPO explore in parallel; as the ants iterate, pheromones are distributed along the exploration paths through iteration, while PPO updates the parameters of the target network based on the loss function.

In the BEAGM-PPO algorithm, time-varying action selection weights are employed to modify the action selection policy. The BEAGM-PPO algorithm preserves the agent’s random exploration of the environment. Thereby, it allows the agent some leeway to explore the environment and address the issue where the ACO algorithm can become trapped in local optima due to its positive feedback mechanism. The figure below shows the exploration rate curves for the three exploration methods. During the first 60% of the period, actions are selected using the three exploration methods with certain probabilities, where the sum of the probabilities for ACO and random actions is p(p∈[0,1]). The probability of adopting the ACO decision is $\frac{p}{n}$, while the probability of a random exploration is $p-\frac{p}{n}$. n represents the exploration rate parameter, which is adjusted based on actual training conditions. The probability of using the neural network for decision-making is 1 − p, where p starts at 1 and gradually decreases to 0. This result indicates that, in the early stages, the probability of using ACO and random actions for decision-making is higher; under the guidance and exploration of these methods in the early stages, the network gradually converges. In the middle and late stages, the system gradually shifts to neural network-based decision-making, and by the late stage, after the network has converged, action decisions are made entirely by the neural network.

**Figure 6:**
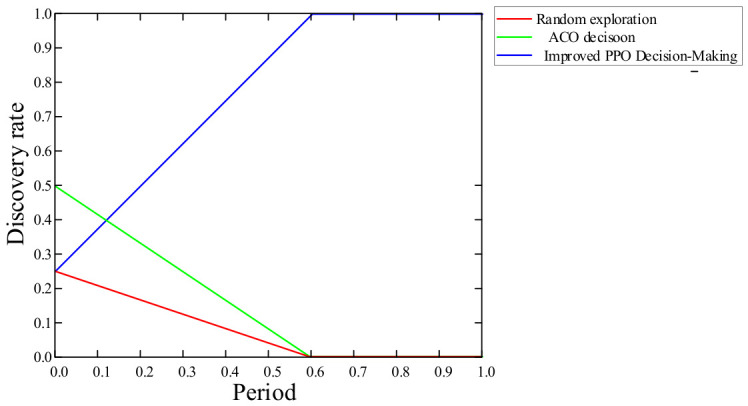
Curve of changes in exploration rate.

The specific switching strategies for the three exploration methods are as follows:

(1) During the early and middle stages of training (the first 60% of the training period), three exploration methods are used in conjunction to select actions. Specifically, the probability of PPO-based decisions is gradually increased, while the probabilities of ACO-guided and random decisions are reduced. The combination of ACO-based guidance and stochastic exploration enables more efficient exploration of globally optimal strategies.
(2) During the latter stages of training (after 60% of the training time has elapsed), decision-making is handled entirely by the PPO network to ensure the algorithm’s stability and the network’s full convergence. It can be seen from Figure 6.

The BEAGM-PPO algorithm employs three strategies for action selection, allowing the agent to perform actions within a controlled range that differ from those of ACO and PPO.

## 4. Simulation Results

To discuss how AGVs may navigate autonomously and avoid obstacles, in this section, the training and validation of the BEAGM-PPO method are presented. The training procedure was split into two parts: training the BEVFormer model and training AGM-PPO using simulation results based on BEVFormer. A simulation environment based on Unity3D was used to evaluate the agents that had been trained using BEAGM-PPO.

Since the proposed approach depends on a reference model, it is necessary to train and evaluate the model separately in advance. The test environment is the same as that used for BEAGM-PPO.

### 4.1. Simulation Environment

A Python 3.8 training environment and a Unity3D testing environment were used in this work. Simulation results based on BEVFormer in the training environment for AGM-PPO training were derived. Images of obstacles taken by AGVs in the test environment were used to train the BEVFormer model. Lastly, a test environment was used to evaluate the BEAGM-PPO-trained agent.

**Figure 7:**
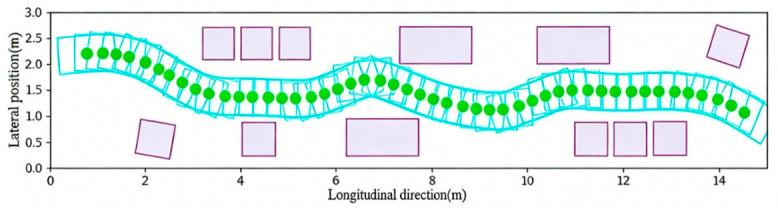
Training environment.

In the scenario described in this work, the AGV speed was set to 1.2 m/s. The maximum steering angular velocity was 0.087 rad/s. The maximum acceleration was set to 0.2 m/s and the maximum path angle (the angle between the velocity vector and the vehicle centerline) was 0.13 rad. When there were pedestrians in the scene, a constant speed of 1.0 m/s was enforced. Pedestrians turned once every 12 s, with the turning angle $\theta_{obs}\in [-\pi /2,\pi /2]$ generated randomly. Because the comfort zone for pedestrians was 0.7 m, the no-fly zone was set at 0.7 m. As can be seen in Figure 7, a 15 × 3 training environment was created using the matplotlib plotting library in Python to train AGM-PPO using the output from BEVFormer. The green indicates the current position of the AGV, while the purple rectangle represents the camera field of view and the safety zone for pedestrians and obstacles.

**Figure 8:**
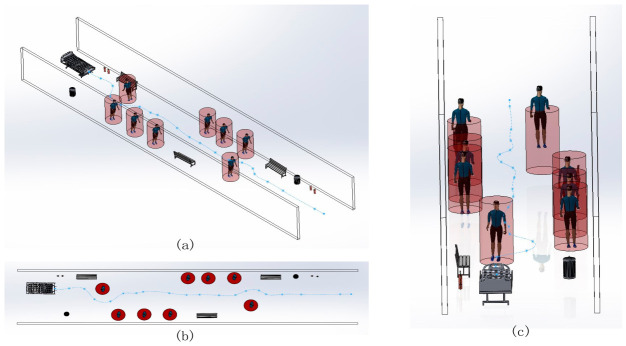
Test environment in Unity3D. (**a**) Side view; (**b**) Bird’s-eye view; (**c**) First-person view.

Since Unity3D offers realistic collision detection capabilities and the ability to simulate in-vehicle cameras, a test environment in Unity3D was created to assess the effectiveness of the BEAGM-PPO suggested in this work. Figure 8 shows a rendering of the test environment. In the test environment, trash cans, fire hydrants, and benches are considered fixed obstacles, while pedestrians’ positions and directions are random. They may be moving or stationary. The red circles around the pedestrians in the image indicate the comfort zone.

### 4.2. Experimental Settings

To ensure fairness in the comparison of different methods and the reproducibility of experimental results, a unified experimental setup and implementation standard for all compared algorithms (including reinforcement learning-based methods such as DQN, PPO, and SAC, as well as improved methods incorporating ACO mechanisms) were established. More specifically, all methods were executed under the same training environment and testing platform, using consistent state spaces, action spaces, and task definitions, and were trained and evaluated based on the same data distribution.

In terms of training protocols, all RL methods were trained for a uniform number of 500 episodes with a fixed interaction step size, and performance was evaluated once the policy converged or stabilized. Regarding hyperparameter settings, DQN, PPO, and SAC all used a discount factor of γ=0.99 to ensure consistent modeling of long-term returns. Specifically, PPO employed a clip ratio (0.2) to limit the magnitude of policy updates; SAC adopts a maximum entropy framework and incorporates an automatic temperature parameter adjustment mechanism; while DQN utilizes a target network and experience replay mechanism to enhance training stability. For the ACO mechanism, the pheromone update strategy was designed in coordination with the reinforcement learning strategy update process to provide global guidance without altering the core structure of the original RL framework, thereby ensuring the fairness of the comparison. In terms of network architecture and initialization, all deep models employ the mainstream fully connected network architecture (two hidden layers, each with 256 neurons) and a consistent activation function (ReLU). A unified parameter initialization method (Kaiming initialization) was used, and the Adam optimizer with a piecewise learning rate was adopted across the board to avoid introducing additional bias due to differences in training details. In terms of implementation and runtime environment, all methods were implemented using the same deep learning framework (PyTorch 2.7.1) and run under consistent hardware and software conditions. The random seed size was set to 50.

### 4.3. Reference Model Training and Results

In Unity3D, 20 different hospital corridor scenes were generated, with initial conditions—such as the placement of vehicles and obstacles—randomly generated at the start of each episode. During testing, an additional 50 hospital scenarios were generated to evaluate the generalization ability of the reference model.

Two human experts used AGVs to complete autonomous navigation and obstacle avoidance tasks in a simulated environment, and their actions were recorded as expert demonstration data. Humans observe their environment from a first-person perspective, as shown in Figure 8c, and perform four distinct actions. Human operators can control the AGV using the keyboard. The AGV speed was set for the longitudinal direction using two separate actions: acceleration and deceleration. Horizontal movement: two discrete actions, turning a certain angle to the left or right. Experts can control both vertical and horizontal movements simultaneously without having to constantly provide input. The AGV will continue to execute the last programmed action until an operator changes the destination. Data from the demonstration was kept in state-action pairs, and only actions that successfully complete a task are stored. Both the action and the state were normalized to [−1, 1] and [0, 1], respectively. Thirty expert-demonstrated trajectories were collected and the effects of the utilized sample size were examined to train the expert imitation policy on AGM-PPO training and testing outcomes.

**Figure 9:**
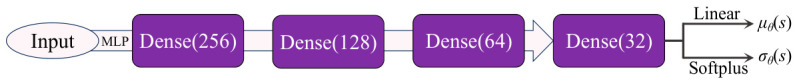
The structure of the uncertainty expert policy model.

Five separate policy networks were trained using different random seeds to create an ensemble-based uncertainty model. Every network in the integrated network was trained for 100 epochs, whether the dataset was shuffled or the network weights were initialized. The policy network in the integration is shown in Figure 9. Directly regressing these discrete values may lead to unstable training behaviour and degraded network performance. As a result, the discrete action was supplemented with a little random number taken from a normal distribution, which is not anticipated to have a significant impact on the actual operation. To create the final action distribution, 0.1 was added to the standard deviation of the Gaussian distribution of the generated uncertainty policy model, making sure that the distribution is sufficiently broad to encompass all potential actions. The setup was executed on an AMD Ryzen 5800X CPU.

### 4.4. BEAGM-PPO Training Experiments

The training of BEAGM-PPO consists of two parts: the first involves training the BEVFormer model, and the second involves training AGM-PPO using simulation results based on BEVFormer, which is the most time-consuming part of the training process. The simulation results based on BEVFormer outputs can effectively reduce training time, as these results are computed much faster than the actual results produced by the BEVFormer model. Furthermore, when the AGV environment changes, only the BEVFormer needs to be retrained. However, based on the simulation results from BEVFormer, the AGM-PPO model does not require retraining. The most time-consuming step in the BEAGM-PPO training procedure is the AGM-PPO training using BEVFormer simulation data.

#### 4.4.1. Training the BEVFormer Model to Extract Obstacle Information

The dataset used in this work consists of corridor scenes captured in complex hospital settings, with multiple shots taken from real-world locations at Jilin Central Hospital and the First Bai Qiuen Hospital of Jilin University in Changchun. The data acquisition device is a stereo camera mounted on a self-built smart transport vehicle. The collected data fully reflects the various real-world conditions within the hospital, including potential obstacles such as people, trash cans, and chairs. An HBVCAM-12M2353 V11 camera (DUO SHI JIE, Guangzhou/Shenzhen, China) with 12 megapixels and a field of view of 85° was utilized. Images were captured at a resolution of 1920 × 1820 and saved in JPG format. In total, 3500 raw images were selected and carefully annotated. The dataset was randomly split into three groups in an 8:2 ratio: a training set and a test set. To prevent overfitting, image augmentation was applied to a subset of the data after clustering, including random cropping, Gaussian noise, horizontal and vertical flipping, and scaling. After augmentation, the dataset grew to 5823 images. In this work, all image inputs were standardized to a size of 640 × 640 × 3. Training was performed using the Adawm algorithm, with an initial learning rate of 0.005, a weight decay factor of 0.0005, a momentum factor of 0.937, and a batch size of 64. The framework was developed in the Anaconda environment and is based on the PyTorch deep learning framework. After obtaining information about the obstacle, triangulation from the stereo cameras was used to convert the object’s coordinates in the image into its distance from the co-axial plane of the stereo cameras [30].

**Figure 10:**
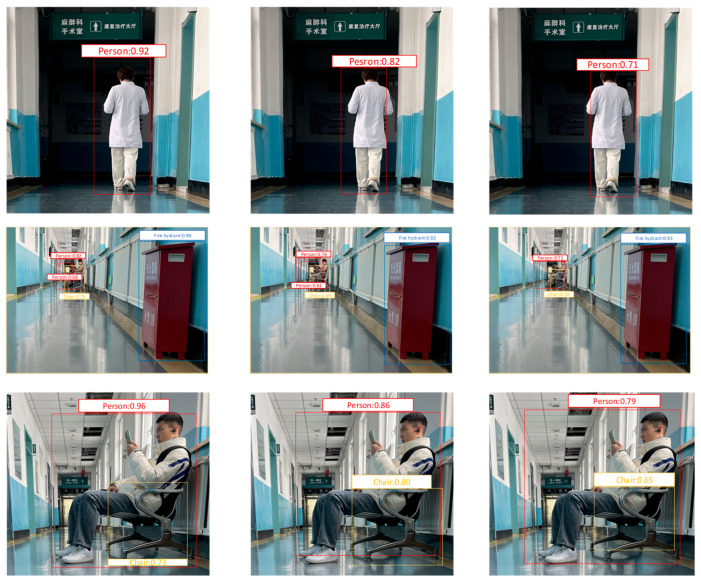
Results from different detection models.

To validate the effectiveness of the BEVFormer model, it was compared with other object detection models. Under identical experimental conditions, the results are shown in Figure 10 and Table 1. In the experiment, three identical images were selected at random. It is clear that BEVFormer achieved a 100% success rate, whereas the YOLO model sometimes fails to detect objects. Compared with Fast RCNN, the BEVFormer model exhibited significantly higher confidence scores, indicating a high degree of confidence in the reliability of the detected objects. Therefore, in this experiment, the BEVFormer model was selected for obstacle detection.

#### 4.4.2. Training AGM-PPO Based on BEVFormer

**Figure 11:**
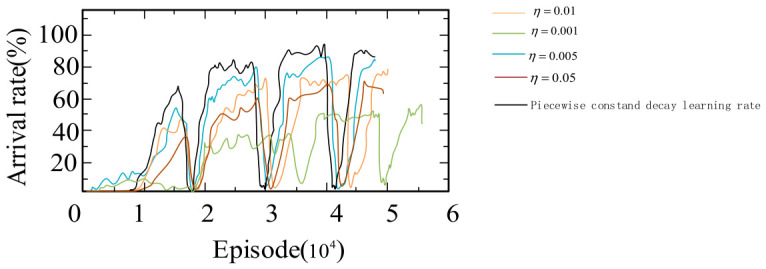
Different Learning Rate Settings.

Since changes in obstacles can lead to excessive time consumption during retraining, AGM-PPO training based on BEVFormer was conducted in the environment shown in Figure 7. Different learning rates and various configuration schemes were applied to select the optimal learning rate that maximizes model performance. The latest 100 episodes’ arrival rate, including the present one, was used to calculate the arrival rate for each episode. As shown in Figure 11, the piecewise constant decay learning rate method achieved the highest arrival rate. Therefore, the network hyperparameter settings are shown in Table 2.

**Figure 12:**
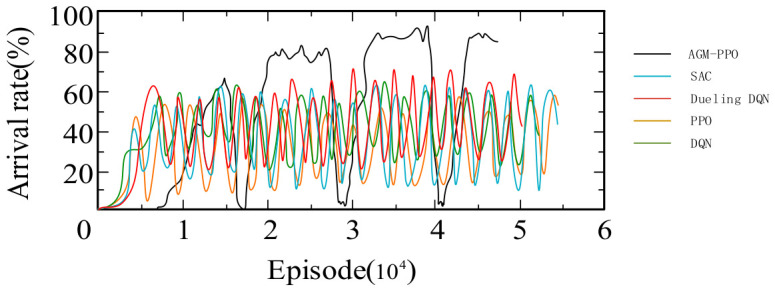
Training curves for different reinforcement learning models.

To verify the effectiveness of the AGM-PPO proposed in this work in a training environment, other baseline models for comparison were selected. The experimental environment and parameter settings for the other baseline models were the same. The network architecture of Dueling DQN consists of 29 × 512 × 128 × 6 and 29 × 512 × 128 × 1 layers. The results are shown in Figure 12. As can be seen, the model proposed in this work achieved the highest arrival rate of 85% compared with other models, eventually converging to 80%. Obstacles affect the random placement of start and goal positions, which can occasionally produce unrealistic navigation conditions. Variations in the success rate can also be caused by elements like barriers that are too near to one another or a short distance between the starting point and the obstacles.

**Figure 13:**
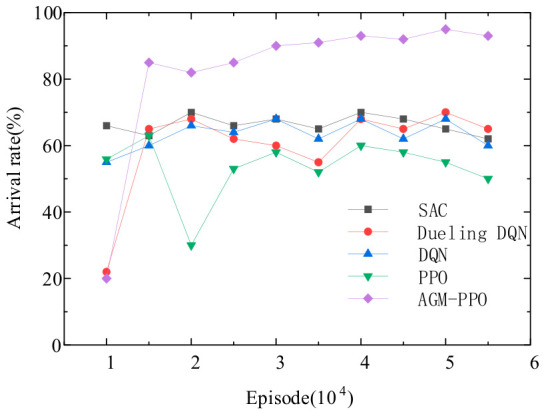
Arrival rates at various phases of training.

Every 5000 training episodes, the trained models were kept to better illustrate the trends in the training outcomes. After training, each model was evaluated for 500 episodes, and the arrival rate was noted. The results are shown in Figure 13. During the first 15,000 episodes of training, the performance of other baseline models was better than that of AGM-PPO. This is because network optimization is performed only during the relevant steps. Every step performed within baseline models is regarded as operationally valid. However, only some of the steps in AGM-PPO are effective. This result indicates that, during the early stages of training, the optimization rate in AGM-PPO does not perform as well as other baseline models. The unequal distribution of different sorts of experiences in the dataset limited alternative baseline models, whereas AGM-PPO arrival rate increased after 15,000 episodes. Other baseline models finally achieved around 60%, and AGM-PPO arrival rate eventually reached about 90% as training progressed. The experiment showed that the AGM-PPO model converges more effectively than other models. Next, after integrating AGM-PPO with BEVFormer, the agent trained using BEAGM-PPO will continue testing in the test environment built within Unity3D.

As shown in Figure 12 and Figure 13, BEAGM-PPO exhibited a low reach rate during the first 15,000 episodes. This is primarily because adaptive priority experience replay filters out high-quality samples early on, reducing the number of steps required for updates while improving sample validity and suppressing interference from low-value experiences. As high-quality experiences accumulate and β gradually increases toward uniform sampling, its performance rapidly improved after 15,000 iterations and converges to approximately 90%, significantly outperforming the baseline model, which stagnates at around 60%. This outcome demonstrates a slow start, fast finish convergence pattern.

### 4.5. Testing Experiments in the Unity3D Environment

In this section, the BEAGM-PPO proposed in this work will be tested in the test environment shown in Figure 8. In this scenario, the input state for the BEAGM-PPO-trained agent was derived from a collection of images the camera took and AGV location data obtained via GPS. It is necessary to test various baseline models in the same settings in order to verify the efficacy of the AGM method. However, since models such as SAC, DQN, Dueling DQN, and PPO lack the ability to detect obstacles, the BEVFormer model was incorporated into these baseline models for testing, as shown in Table 3. In addition, to confirm the BEVFormer output method’s efficacy, BEVFormer was combined with other baseline models and various combinations of YOLO and Fast RCNN were also tested.

#### 4.5.1. Testing with Different Quantities of Immobile Barriers

**Figure 14:**
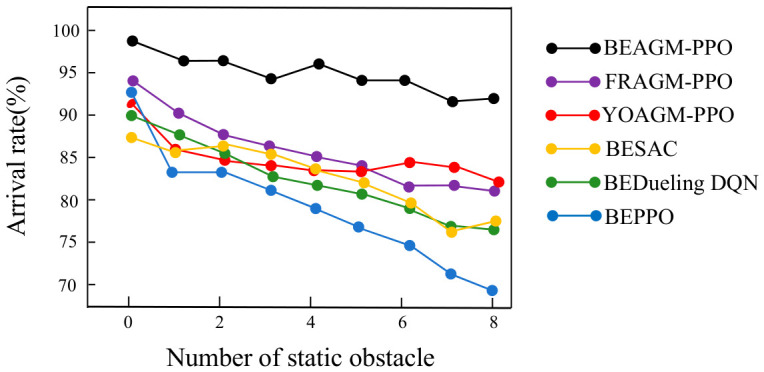
Model achievement rate in environments with varying numbers of static obstacles.

In this experiment, the agents trained using the models built from the various methods described in the previous section were tested in an environment containing multiple static obstacles. In each different scenario, the number of static obstacles increased from 1 to 8. For each scenario, the number of obstacles and test agents trained was specified using different methods for 500 episodes. In each episode, the starting and ending points of the AGV were randomly generated. In Figure 14, the test results were displayed.

As shown in Figure 14, in scenes containing multiple static obstacles, the agent trained using BEAGM-PPO achieved the highest arrival rate, significantly outperforming other models, while also exhibiting the lowest dropout rate. The arrival rate of agents trained with BEAGM-PPO decreased from 98.1% to 94.2% as the number of static barriers increased. Changes in the arrival rates of other models are shown in Table 4. Compared with agents trained using other models, agents trained using BEAGM-PPO were less affected by changes in the number of obstacles. As the number of static obstacles increased, when there were two or more obstacles, all agents began to experience varying degrees of decline. The BEAGM-PPO, FRAGM-PPO, and YOAGM-PPO approaches outperformed other methods that do not use AGM because they do incorporate the AGM method suggested in this work. In terms of detection models, since BEVFormer achieved better detection results than Fast RCNN and YOLO, BEAGM-PPO exhibited a higher hit rate than the FRAGM-PPO and YOAGM-PPO methods. As a result, in situations with static impediments, the agent trained with the BEAGM-PPO approach presented in this work yielded the best performance.

It is worth noting that, as shown in the Change column of Table 4, while the number of static obstacles increased from 1 to 8, the arrival rate of BEAGM-PPO decreased by only 3.3%, whereas BEPPO saw a significant drop of 23.8% and BEDueling DQN decreased by 12.1%. This result indicates that BEAGM-PPO exhibits significantly greater robustness to variations in the number of static obstacles. The stable detection capabilities of BEVFormer, combined with the global guidance provided by the ACO pheromone, are the primary reasons for its consistent performance.

#### 4.5.2. Testing with Various Moving Obstacle Counts

**Figure 15:**
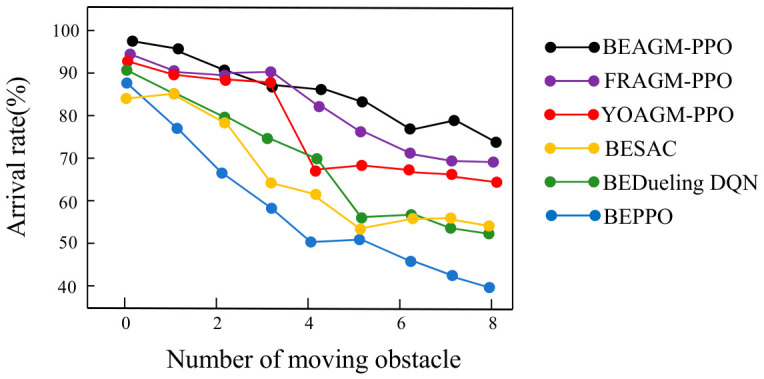
Model attainment rates in settings with various moving obstacle counts.

In this experiment, the same model used in the previous experiment was tested in an environment containing multiple moving obstacles. The scenario for this experiment was similar to that of the static obstacle test. In each different scenario, the number of moving obstacles increased from 1 to 8. The number of obstacles for each different scenario was specified. Test agents trained using different methods for 500 episodes each. The obstacles and the beginning and finishing of AGV positions were chosen at random for every episode. Figure 15 displays the test findings. The agent trained using BEAGM-PPO outperforms the other agents in terms of arrival rate, as seen in Figure 15. The arrival rates for the different methods are shown in Table 5.

As shown in Table 5, the arrival rate in this experiment slightly decreased as the number of obstacles increased. This is because moving impediments are harder to avoid. Similar to the static experiments, agents trained using the AGM method achieved a higher arrival rate. Among the methods that incorporate AGM, the method using BEVFormer for model detection achieved a higher hit rate than Fast R-CNN and YOLO. Consequently, the BEAGM-PPO exhibited enhanced performance under dynamic obstacle conditions.

Compared with static scenarios, the introduction of dynamic obstacles caused a more significant drop in the arrival rates of all models, which is consistent with the expectation of higher uncertainty in dynamic environments. However, as shown in Table 5, the decline in the arrival rate of BEAGM-PPO (18.9%) remained significantly lower than that of BEDueling DQN (36.9%) and BEPPO (48.1%). This advantage is primarily attributed to the reference model’s dual modelling of policy uncertainty and model uncertainty, which enables the agent to maintain relatively stable decision outputs when facing out-of-distribution states—such as random pedestrian movements—rather than exhibiting severe policy fluctuations.

#### 4.5.3. Testing with Different Quantities of Mixed Obstacles

**Figure 16:**
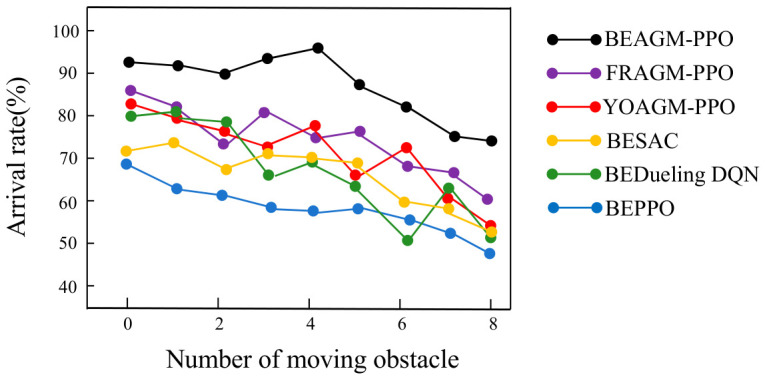
Model arrival rate in mixed obstacle environments.

In this experiment, the agents, trained using the same model-based methods as in the previous two experiments, were put to the test in the environment with several static and moving obstacles. There were eight obstacles in total in each scenario, and the number of moving obstacles increased from 0 to 8 in a consecutive manner. All agents were tested over 500 episodes and each scenario had a certain amount of static and dynamic obstacles. The beginning and finishing locations of AGV and the obstacles were chosen at random for each test. Figure 16 displays the outcomes. In the test environment with mixed obstacles, since there were a total of 12 obstacles, the arrival rate began to decline to varying degrees as the quantity of moving obstacles rose, particularly for the YOAGM-PPO. Due to the impact of YOLO performance, a significant drop occurred when the number of moving objects increased to three. Similar to the previous two experiments, Figure 16 shows that the combination of the AGM method and the BEVFormer model achieved the best performance. The reach rates for the different methods are shown in Table 6. Based on the results of these three experiments, it can be concluded that the agents trained using the BEAGM-PPO method proposed in this work were capable of effectively performing autonomous navigation and obstacle avoidance.

A comprehensive analysis of the performance metrics across the three sets of experiments leads to a consistent conclusion: under static, dynamic, and mixed obstacle interference scenarios, the drop in the arrival rate of BEAGM-PPO remains consistently lower than that of all other comparison methods. This cross-scenario stability advantage indicates that the proposed method is not merely optimized for specific types of obstacles. However, it rather fundamentally enhances the agent’s robustness against various types of environmental disturbances through the synergistic interaction of the BEVFormer’s perception reliability, the uncertainty reference model, and ACO-guided global exploration.

**Figure 17:**
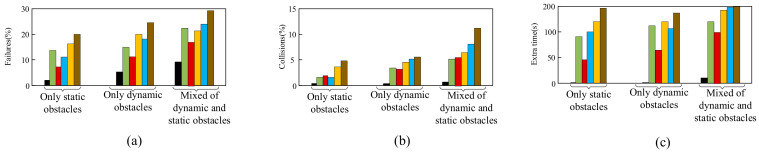
Experimental results of different models under various evaluation metrics. (**a**) Failures; (**b**) Collisions; (**c**) Extra time.

As shown in Figure 17, BEPPO exhibits a relatively high probability of failing to respond to obstacles in a timely manner, leading to collisions during navigation. In contrast, BEAGM-PPO maintains a stable trajectory throughout the process and successfully reaches the target. This result further demonstrates that BEVFormer provides more reliable detection performance compared with YOLO and Fast R-CNN. Meanwhile, the uncertainty-aware guidance from the reference model enables the agent to adopt more conservative and safer decisions when facing perception noise, rather than triggering cascading decision errors due to occasional misdetections.

Combined quantitative and trajectory visualization analysis results indicate that the effectiveness of BEAGM-PPO emerges from the collective influence of four key components: BEVFormer-based perception, the uncertainty-aware reference model, ACO-guided exploration, and adaptive experience replay. These modules jointly enhance the method from four complementary aspects, namely perception reliability, policy stability, exploration efficiency, and sample utilization, thereby supporting the overall superiority of the proposed approach.

#### 4.5.4. The Impact of Uncertainty Policies and Integrated Models

The integrated model, which quantifies the uncertainty of both the policy and the model to derive an expert policy, was presented in the preceding section. In this experiment, other methods were used to validate the integrated model—the Reference Model—composed of the proposed uncertainty policy. Using just one policy network from the integrated model, a Gaussian distribution policy that just takes policy uncertainty into account. Another approach is to use pre-trained large language models such as LLaMA [44].

**Figure 18:**
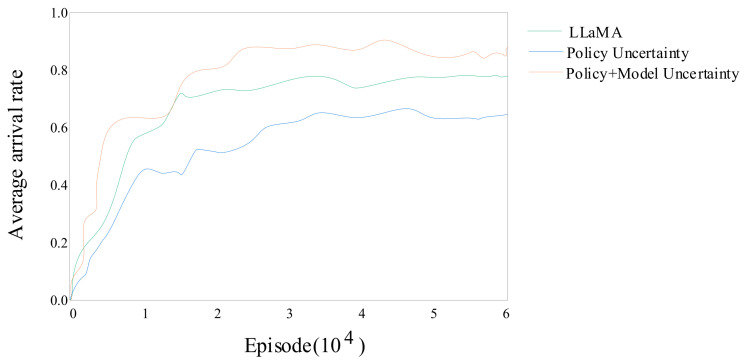
Comparative tests of different reference models.

As shown in Figure 18, the AGM-PPO model proposed in this work benefits from the integrated uncertainty policy. In particular, integrated methods can stabilize the process of training, thereby achieving a higher arrival rate. A single Gaussian policy network might not be able to offer a decent search space and account for uncertainty in uncertain situations. However, when using the LLaMA model as a reference model, it does not acquire knowledge related to navigation and obstacle avoidance through imitation learning from expert demonstrations or through reinforcement learning in a simulated environment.

#### 4.5.5. Expert Demonstration of the Impact of Sample Size

The quantity of expert demonstration data has an impact on AGM-PPO training quality as well. To this end, the influence of different sample sizes on the final test results was examined. The expert demonstration for uncertainty was trained in this work using example trajectories of 10, 20, and 30. Then, the policy was used to train an integrated model to guide the agent training.

**Figure 19:**
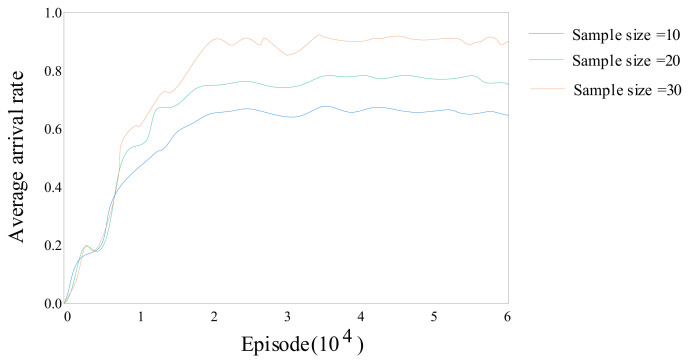
Effect of different sample sizes on the average arrival rate.

The results in Figure 19 show that as the number of expert demonstration samples increased from 10 to 30, the average reach rate of AGM-PPO continued to improve, and the learning rate also accelerated. This phenomenon can be explained from two perspectives. First, more demonstration data enables the reference model to cover a broader distribution of the state space, thereby providing effective action guidance to the agent in more scenarios and reducing blind exploration in sparse reward regions during reinforcement learning. Second, when training data is abundant, the uncertainty estimation module can output expert policy distributions with higher confidence and lower variance, making the guidance provided by the KL divergence constraint on policy updates more precise and thereby improving the efficiency of each experience’s contribution to policy optimization. This result indicates that the performance ceiling of BEAGM-PPO is closely related to the quality and quantity of expert demonstration data; in practical deployment, appropriately expanding the high-quality demonstration dataset can further enhance system performance.

#### 4.5.6. Comparison of Related Models

The results of the comparison between the different models are shown in Table 7. Specifically, although the proposed framework integrates several existing techniques, its contribution does not lie in a simple combination of modules. Instead, the main novelty is reflected in the following aspects.

BEVFormer-based perception is integrated into the RL navigation framework to provide structured bird’s-eye-view environmental representations, enabling more robust obstacle localization and spatial awareness in complex hospital corridors.

An ACO-inspired guidance mechanism is incorporated into PPO-based reinforcement learning to reduce inefficient random exploration during early training, thereby improving convergence efficiency and navigation stability.

A reference-model-guided uncertainty-aware decision strategy is introduced, where expert demonstrations are not directly used for behavior cloning, but instead dynamically guide the agent under uncertain states, improving adaptability and robustness compared with conventional imitation-RL methods.

An adaptive prioritized replay mechanism is designed to optimize sample utilization by emphasizing high-value experiences during training, further enhancing learning efficiency and policy stability.

Unlike many existing vision-based navigation approaches that primarily focus on collision avoidance, the proposed method additionally considers pedestrian comfort and socially aware navigation in hospital environments.

#### 4.5.7. Analysis of Typical Incidents

Based on the test experiments, and in order to more clearly and definitively validate the effectiveness of the AGM-PPO-trained agent, typical events that occurred during the testing process were analysed. For example, the process by which an AGV performs tasks in an environment with multiple dynamic obstacles and the process by which it performs tasks in an environment containing a mix of dynamic and static obstacles. In Figure 19, the colours of the paths controlled by the agent for the AGV are shown in Table 8.

**Figure 20:**
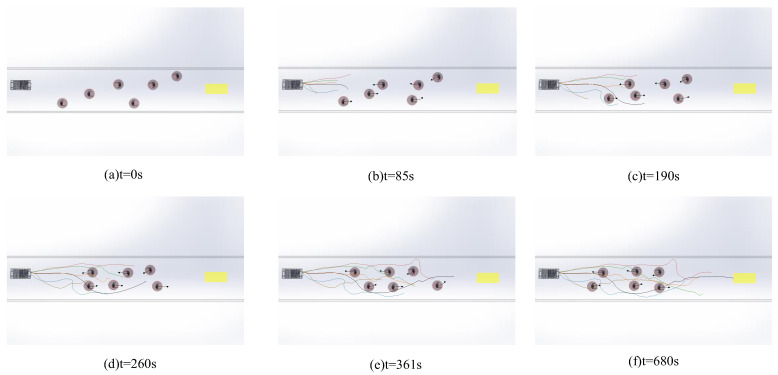
AGV trajectories in scenarios with dynamic obstacles.

Figure 20 shows the test results for the agent in an environment with multiple dynamic obstacles. In the figure, the gray circles around the pedestrians represent their comfort zones, while the gray rectangle on the left represents the AGV, which also marks the starting point. The yellow rectangle on the right represents the AGV destination. The black arrow indicates the pedestrian’s direction of movement. Figure 20 shows the paths taken by AGVs using different algorithms as they travel toward their destinations. Figure 20a shows the starting point of this experiment, where the starting point and destination of the AGV, as well as the initial positions of the pedestrians, have been set. As can be seen in Figure 20c, all AGVs moved in the direction of their destinations throughout the test first phase. Agents were taught using various methods demonstrated distinct obstacle-avoidance policies when faced with dynamic impediments. The AGV controlled by the agent trained using the AGM-PPO approach proposed in this research reached its target, as illustrated in Figure 20f. None of the AGVs controlled by other models have arrived. Furthermore, the YOAGM-PPO algorithm exhibited significant path detours, with path lengths being significantly longer than those of the AGM-PPO algorithm. Therefore, in a testing environment with dynamic obstacles, the AGM-PPO method proposed in this work outperformed the other five methods listed in Table 7.

**Figure 21:**
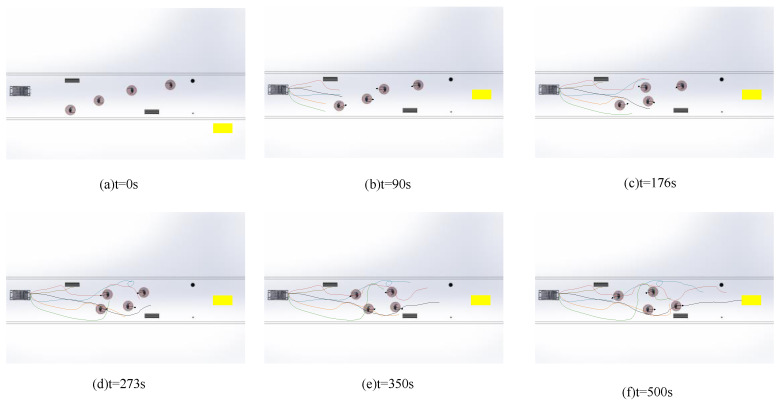
AGV trajectories in mixed obstacle scenarios.

Tests in environments containing both static and dynamic obstacles were also conducted and the results are shown in Figure 21. Similar to the dynamic environment experiment, measurements were taken at three different time points. The colours of the different AGV paths in the figure correspond to those in Table 7. The two black rectangles in the image represent corridor seating; the small black circles represent trash cans; and the small black dot in the lower right corner represents a fire hydrant. As shown in Figure 21b, the agents trained using all methods steer the AGV toward the destination. However, the BE-PPO method failed to avoid the obstacle in time while driving, resulting in a collision and mission failure. As shown in Figure 21d, the motion path of the YOAGM-PPO becomes distorted due to misidentification. At t = 500 s, only the BEAGM-PPO method successfully reached the destination. Therefore, agents trained using the BEAGM-PPO method performed better in environments containing both static and dynamic obstacles.

Based on the above experiments, the BEAGM-PPO method proposed in this work demonstrates significant performance improvements compared with other agents and is capable of autonomous navigation and obstacle avoidance.

## 5. Discussion

It should be noted that a simplified two-dimensional Python environment was employed during the training phase, while for the testing phase, a Unity3D environment equipped with a real-world physics engine and visual modelling capabilities was utilized. This setup inevitably introduces a simulator gap. This gap is primarily manifested in the representation of the environment (discrete 2D vs. continuous 3D), the source of perceptual input (simulated features vs. camera images), and the complexity of dynamic interactions. From a reinforcement learning perspective, this mismatch between the state distribution and observation noise may lead to a decline in policy performance during transfer, particularly when dealing with out-of-distribution states, where traditional methods are prone to producing unstable decisions.

Nevertheless, the method proposed in this work mitigates the aforementioned issues through a multi-level mechanism. First, the training phase does not rely directly on idealized states; instead, it performs policy learning based on the perception results output by the BEVFormer. This introduces perception errors and uncertainty to some extent, aligning the training distribution with the true input distribution in the testing phase. Second, by modelling both policy uncertainty and model uncertainty in the reference model, the policy maintains a reasonable action distribution even in unseen states, thereby enhancing cross-domain robustness. Furthermore, the introduction of the ACO pheromone mechanism provides global guidance based on accumulated historical experiences, helping to maintain decision stability when the environment changes. At the same time, adaptive prioritized experience replay enhances the policy’s generalization ability to complex scenarios by reinforcing learning from key samples.

The experimental results further validate the effectiveness of the proposed design. In the Unity3D testing environment, the proposed method outperformed the baseline method across various obstacle configurations, and its performance degradation was relatively minor as environmental complexity increased. This result indicates that, despite certain differences in the simulation domains, the learned strategy still demonstrates strong cross-environment transferability. However, it should be emphasized that this method has not yet fundamentally eliminated the domain gap between simulators. Future work will explore strategies such as domain randomization, cross-domain consistent learning, and online fine-tuning in real-world environments to further enhance the model’s generalization and reliability in complex real-world settings.

Future work will focus on bridging these gaps by deploying the proposed framework on real AGV platforms for empirical validation in real hospital environments. In particular, sim-to-real transfer techniques, such as domain randomization and domain adaptation, will be investigated for the improvement of generalization across environments. Moreover, incorporating multi-sensor fusion (e.g., combining vision with LiDAR or depth sensors) is expected to enhance perception robustness under challenging conditions. Finally, integrating safe reinforcement learning mechanisms and expanding the scale and diversity of expert demonstrations may further improve policy reliability, stability, and real-world applicability.

## 6. Conclusions

To address the autonomous navigation and obstacle avoidance capabilities of AGVs in hospitals, a BEAGM-PPO method was proposed in this work. First, during the expert assessment phase, experts make preliminary estimates of task performance based on their own preferences. Subsequently, using the experts’ demonstration data, expert policies are derived through imitation learning and uncertainty estimation, and these are used as a reference model to guide the action outputs in the reinforcement learning algorithm. In terms of the model, this method incorporates the BEVFormer model to enhance image detection capabilities. The pheromone mechanism of ACOs and the adaptive importance-weighted experience replay mechanism were also introduced to determine the selection of optimal actions, thereby further improving training performance. Finally, based on pedestrian comfort, a reward function was proposed to effectively avoid pedestrian obstacles. The simulation results show that the proposed method not only achieves optimal performance but also successfully navigates around obstacles to reach the finish line, outperforming the baseline algorithm.

Additional functionalities, such as voice control and 3D reconstruction, are required to further improve the motion trajectories of mobile robots. These directions define the future research roadmap of our group. A thorough awareness of the robot’s surroundings and the capacity to instantly adjust to changing circumstances are necessary to implement these extra control features. To manage the complexity of dynamic settings and guarantee the security and effectiveness of mobile robots, strong and dependable algorithms must be developed. The capacity of mobile robots to successfully navigate difficult areas and adjust to changing situations in real time is also considered crucial, as they grow more prevalent in a variety of sectors.

## Figures and Tables

**Table 1:** Detection results for different models.

Image Number	BEVFormer			Fast RCNN			YOLO		
	Success in Detecion or Not	Confidence Coeffcient	FPS	Success in Detecion or Not	Confidence Coeffcient	FPS	Success in Detecion or Not	Confidence Coeffcient	FPS
1	Success	0.92	47.30	Success	0.82	28.61	Success	0.71	68.52
2	Success	0.81	49.12	Success	0.74	30.35	Failure		
3	Success	0.85	48.53	Success	0.83	27.45	Success	0.72	67.45

**Table 2:** Hyperparameter Tuning for BEACO-PPO.

Hyperparameter	Value
Batch Size	64
Replay memory size	100,000
Discount factory γ	0.99
Clip ratio	0.2
Entropy coefficient	0.5
Piecewise learning rate [$\eta_{1},\eta_{2},\eta_{3},\eta_{4}$]	[0.001, 0.005, 0.0001, 0.00005]
Number of Layers	3
Hidden Size	1024
Activation Function	ReLU
Dropout Rate	0.5

**Table 3:** Statistical table of test methods composed of different model combinations.

Agent	Contained AGM Method or Not	Contained Object Detection Method
BEAGM-PPO	Yes	BEVFormer
YOAGM-PPO	Yes	YOLO
FRAGM-PPO	Yes	Fast R CNN
BESAC	No	BEVFomrer
BEDueling DQN	No	BEVFormer
BEPPO	No	BEVFomer

**Table 4:** Arrival Rates in an Environment with Static Obstacles.

Agent	Achievement Rate When There Are the Fewest Obstacles (%)	Arrival Rate When There Are the Most Obstacles (%)	Change (%)
BEAGM-PPO	98.1	94.8	3.3
FRAGM-PPO	94.8	83.7	11.1
YOAGM-PPO	91.0	84.4	6.6
BESAC	82.5	79.1	3.4
BEDueling DQN	90.3	78.2	12.1
BEPPO	93.7	69.9	23.8

**Table 5:** Arrival Rates in Environments with Dynamic Obstacles.

Agent	Achievement Rate When There Are the Fewest Obstacles (%)	Arrival Rate When There Are the Most Obstacles (%)	Change (%)
BEAGM-PPO	97.6	78.7	18.9
FRAGM-PPO	93.8	74.2	19.6
YOAGM-PPO	91.2	69.8	21.4
BESAC	83.2	57.3	25.9
BEDueling DQN	90.1	53.2	36.9
BEPPO	89.7	41.6	48.1

**Table 6:** Arrival rates in mixed obstacle environments.

Agent	Achievement Rate When There Are the Fewest Obstacles (%)	Arrival Rate When There Are the Most Obstacles (%)	Change (%)
BEAGM-PPO	92.8	76.2	16.6
FRAGM-PPO	89.4	65.6	23.8
YOAGM-PPO	83.0	57.4	25.6
BESAC	71.1	56.9	14.2
BEDueling DQN	80.2	55.3	24.9
BEPPO	69.6	48.0	21.6

**Table 7:** Comparison of Results from Different Relevant Models.

Model	Average Delivery Rate (%)
Imitation-RL [36]	53.2
ACO-RL [45]	55.1
Uncertainty-aware imitation learning [46]	62.8
vision-based RL navigation [47]	70.7
BEAGM-PPO	76.2

**Table 8:** The agent controls the color of the AGV’s path.

Agent	Path Color
BEAGM-PPO	Black
FRAGM-PPO	Red
YOAGM-PPO	Blue
BESAC	Green
BEDueling DQN	Yellow
BEPPO	Brown

## Data Availability

The raw data supporting the conclusions of this article will be made available by the authors on request.
